# Gender composition mediates social facilitation effect in co-action condition

**DOI:** 10.1038/s41598-017-15437-y

**Published:** 2017-11-08

**Authors:** Na Liu, Ruifeng Yu, Lindong Yang, Xuelian Lin

**Affiliations:** Department of Industrial Engineering, Tsinghua University, Beijing, 100084 China

## Abstract

Working with co-actors is a common work-organization mode. Whether the presence of opposite-sex co-actors (OCs) can induce social facilitation effect and how an actor’s performance is influenced by the gender composition of co-actors remain unknown. The present study aims to examine the influence of the gender composition of co-actors on the intensity of the social facilitation effect. In Experiment 1, participants performed visual search tasks alone and in six co-action conditions with varying gender compositions. In Experiment 2, the participants performed modular arithmetic tasks in three conditions with electroencephalogram activity recorded and salivary cortisol measured: alone, with a same-sex co-actor (SC), and with an OC. Results indicated that the social facilitation effect was stronger in the presence of OCs than in the presence of only SCs. The intensities of social facilitation effect resulting from the varying gender composition of co-actors were obtained and compared. A participant’s power of alpha band was lower, whereas power of beta band and normalised cortisol level were higher in the presence of an OC than in the presence of an SC. These findings provide insights into the influencing mechanisms of gender composition on the intensity of the social facilitation effect in the co-action condition.

## Introduction

Working with co-actors is a common work-organization mode. Co-actors refer to people who perform tasks independently but alongside each other. Given their close physical proximity, co-actors can have a direct perception of each other’s work status and task performance^1^. Working with co-actors (i.e., co-action) occurs in various activities. For example, in real-world baggage-screening tasks at airports and train stations, two or more security screeners perform their individual screening tasks independently in close physical proximity to each other. Given their proximate physical distance, they can directly perceive each other’s work status and performance.

Social facilitation effect, the performance enhancement or impairment that occurs when individuals complete a task in the presence of others, is an interesting phenomenon in the field of social psychology^2^. The presence of others can come in the form of audience, co-actors and virtual agents^3–5^. Previous studies have revealed that the performance of individuals when working alone differs from that when working with co-actors, i.e., the presence of co-actors can enhance the performance of easy or simple tasks and inhibit the performance of difficult or complex tasks^6^. Most theories (i.e., drive theory^7^, self-presentation theory^8,9^, objective self-awareness theory^10,11^ and distraction–conflict theory^12,13^) attribute the mechanisms of the social facilitation effect to individuals’ increased arousal level. The increased arousal enhances the emission of dominant responses (i.e., a correct/quick solution for easy or simple tasks, as opposed to a false/slow solution for difficult or complex tasks)^7^. Arousal is a key concept in social facilitation theories. Some studies described it from the physiological aspects and defined it as the intensity dimension of physiological alertness, readiness and responses^14,15^. Some studies related it to the intensity of psychological activation^16,17^. Others suggested that arousal referred to the degree of a general physiological and psychological activation at a particular moment^18–21^. In short, arousal is a person’s state or intensity of being physiologically and psychologically activated. The causes of the increased arousal of individuals resulting from social presence (e.g., presence of co-actors or audience) have been proposed, such as uncertainty^22,23^, evaluation apprehension^24,25^, self-presentation^8,9^, self-awareness^10,11^ and distraction–conflict^12,13^.

The aforementioned theories are presented from the psychological perspective. As physiological data can be used as indicators of arousal^26^, physiological tests need to be performed to verify the impact of social presence on arousal. In the context of audience paradigm, some attempts have been made to examine the relationships between behavioural performance measures and physiological arousal measures, such as palmar sweat^27^, muscle tension^28,29^, skin conductance^30^, heart rate^30^, respiration rate^30^, cardiac response and vascular resistance^31^. However, few studies have been conducted on how physiological measures change in the co-action condition. As the audience presence is different from the co-action condition to a certain extent, the existing results in the audience-present condition may not be tenable in the co-action condition. Ongoing brain electrical activity measured via electroencephalogram (EEG) reflects functional brain states^32–34^. As an electrophysiological monitoring method to record electrical activity of the brain, not only can EEG be used to measure arousal level^35^ but also directly reflect the activated brain areas, and thus provide us an individual’s neural bases and brain functioning mechanisms in response to the social presence. EEG indicators of arousal include the power of alpha and beta bands through EEG power spectrum analysis, that is, alpha power is negatively correlated with arousal level^33,36–38^, and beta power is positively linked to arousal^39,40^. In addition, salivary cortisol can reflect the individuals’ stress, anxiety and arousal, that is, high cortisol levels are associated with high stress, anxiety and arousal^41–43^. Thus, EEG activities and salivary cortisol will be used to measure individual arousal in the present study.

Previous co-action studies regarding the social facilitation effect involve either all-male or all-female subjects, and have reported that the presence of same-sex co-actors could induce the social facilitation effect^44,45^. However, no study has yet investigated the influences of gender composition on the intensity of the social facilitation effect in the co-action condition. The effect of gender composition has been investigated in the context of the work-organization mode of teams. The results revealed that gender composition can modulate overall team performance. A three-female team can achieve better results than all other gender compositions for three-person teams in a large commercial game^46^. The team performance of equal gender proportion (50% males and 50% females) is optimal in terms of sales and profits in a business field study^47^. Gender composition also influences group decision making; groups are more generous and equalitarian when women are the majority^48^. However, team members in the aforementioned study cooperate and communicate with each other to achieve a common goal^49^. By contrast, co-actors are simply in close physical proximity to each other and perform their tasks independently. They do not cooperate with each other. Thus, findings about the effect of gender composition on performance in a team cannot be directly applied to the co-action condition. Whether and how an actor’s behavioural performance will change with varying gender compositions of co-actors remain unclear. To address these questions, the present study aimed to investigate the effect of the gender composition of co-actors on the behavioural performance of individuals and to reveal its influencing mechanisms.

Two experiments were conducted in the present study. In the first experiment, co-action conditions with varying gender compositions were designed to examine the effects of the presence of co-actors with different gender compositions on the task performance of actors. A basic visual search task was adopted. Experiment 1 had a mixed design. The independent variables were task difficulty (easy, difficult), social presence (alone, co-action), gender (male, female) and gender composition (one male and two females [1M2F], two males and one female [2M1F], three males [3 M], three females [3 F], one male and one female [1M1F] and two males and two females [2M2F]). Previous studies usually utilized a group of three persons because three-person group could represent the male-majority or female-majority situations^46,48^. The 1M1F and 2M2F were also used here as the control groups. Gender and gender composition were between-subject variables, and the remaining variables were within-subject variables. The dependent variable was search performance, measured by response time (RT, milliseconds) and accuracy rates (ARs). Participants were assigned to work in one of six co-action conditions with consideration of both gender composition and performance. The results indicated that the presence of co-actors with all gender compositions can induce social facilitation effect; the gender composition of co-actors mediated the intensity of the social facilitation effect. The effect was stronger when actors performed visual search tasks in the presence of opposite-sex co-actors (OCs) than in the presence of same-sex co-actors (SCs).

Despite the ubiquity of the social facilitation effect across tasks, its neural bases for humans are currently unknown^6,23,50^. In the second experiment, we attempted to provide neurophysiological evidence and address this issue by using EEG recordings and salivary cortisol, that is, whether social presence can increase the arousal level of actors and whether different gender co-actors can induce different arousal levels and thus result in varying intensities of the social facilitation effect. Experiment 2 involved a modular arithmetic task. This experiment had a within-subject design with task difficulty (easy, difficult) and social presence (i.e., alone, working with an SC and working with an OC) as independent variables. The dependent variables were performance measures (i.e., RT and ARs), normalised salivary cortisol level and EEG data, i.e., mean powers of theta, alpha and beta bands in frontal, temporal, central, parietal and occipital lobes. The results of Experiment 2 indicate that working with a co-actor could increase an actor’s arousal level, and the presence of an OC could result in greater arousal than the presence of an SC.

## Results

### Experiment 1: Gender Composition of Co-Actors Mediates the Social Facilitation Effect in Coaction Condition

**Figure 1:**
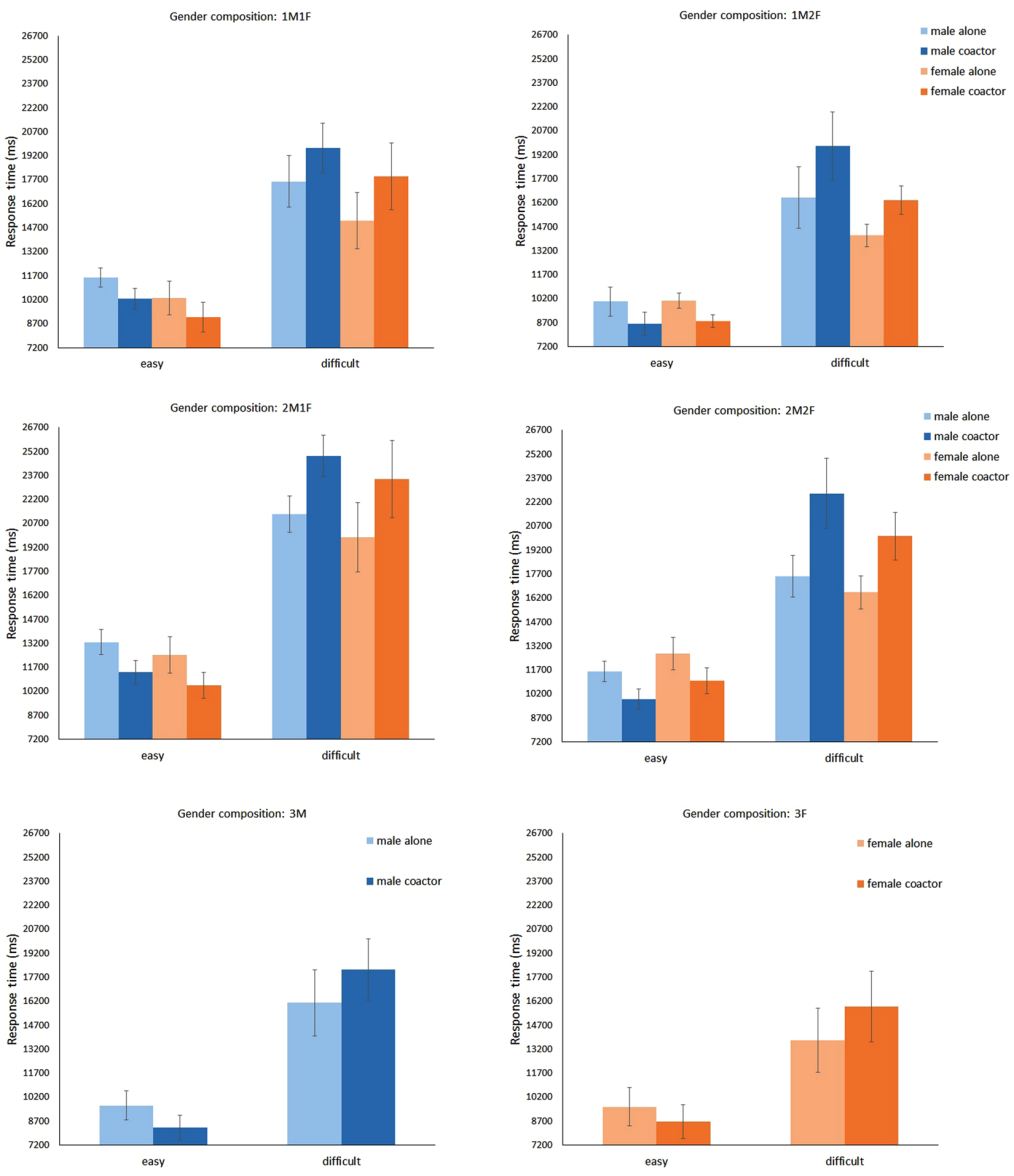
RT as a function of task difficulty and social presence for males and females (Experiment 1). Error bars represent ± 1 SE.

Performance data of male and female participants were separately analysed in each gender composition condition. Male participants were assigned to five co-action groups in correspondence with different gender compositions of co-actors, namely, 1M2F, 2M1F, 3 M, 1M1F and 2M2F. The effects of social presence (*F* (1, 67) = 19.29, *P* < 0.0001) and interaction between task difficulty and social presence (*F* (1, 67) = 154.65, *P* < 0.0001) on RT were significant. Generally, compared with the working-alone condition, males responded faster (slower) in the co-action conditions when performing easy (difficult) search tasks (for easy search tasks, *M*_alone_ = 11,573, *M*_coaction_ = 9,974, *t* (71) = 10.42, *P* < 0.0001; for difficult search tasks, *M*_alone_ = 18,388, *M*_coaction_ = 21,693, *t* (71) = 10.42, *P* < 0.0001). The effects of social presence (*F* (1, 67) = 0.16, *P* = 0.69) and interaction between task difficulty and social presence (*F* (1, 67) = 0.14, *P* = 0.71) on ARs were not significant. Results indicated that the presence of co-actors could induce the social facilitation effect on RT but not on ARs. The performance data of male participants were then separately analysed in each gender composition condition. Social facilitation effect was observed in all five co-action groups (Fig. 1), but its intensity was contingent on the gender composition of the co-actors. The effect size of the interaction between task difficulty and social presence on RT was largest in the 2M2F condition (*F* (1, 11) = 36.19, *P* < 0.0001, η^2^_G_ = 0.129), followed by 2M1F (*F* (1, 23) = 46.55, *P* < 0.0001, η^2^_G_ = 0.073), 1M2F (*F* (1, 11) = 69.56, *P* < 0.0001, η^2^_G_ = 0.048), 1M1F (*F* (1, 11) = 62.24, *P* < 0.0001, η^2^_G_ = 0.044) and 3 M (*F* (1, 11) = 24.97, *P* = 0.0004, η^2^_G_ = 0.028). These indicate that the intensity of social facilitation effect was strongest in 2M2F, and then in 2M1F, 1M2F, 1M1F and 3 M, in strong-to-weak order.

Similar results were observed for female participants. The female participants were assigned to five co-action groups, namely, 1M2F, 2M1F, 3 F, 1M1F and 2M2F. The effects of social presence (*F* (1, 67) = 26.54, *P* < 0.0001) and interaction between task difficulty and social presence (*F* (1, 67) = 163.58, *P* < 0.0001) on RT were significant. Females responded faster (slower) in the co-action conditions than in the working-alone condition when performing easy (difficult) search tasks (for easy search tasks, *M*_alone_ = 10,880, *M*_coaction_ = 9,494, *t* (71) = 9.72, *P* < 0.0001; for difficult search tasks, *M*_alone_ = 15,598, *M*_coaction_ = 18,340, *t* (71) = 10.41, *P* < 0.0001). The main effect of social presence (*F* (1, 67) = 1.94, *P* = 0.17) and the interaction between task difficulty and social presence (*F* (1, 67) = 0.17, *P* = 0.68) on ARs were insignificant. Similar to the case of male participants, the presence of co-actors could induce the social facilitation effect on RT rather than on ARs among female participants. The effect size of the interaction between task difficulty and social presence on RT reveals that the intensity of the social facilitation effect was strongest in the 2M2F condition (*F* (1, 11) = 24.45, *P* = 0.004, η^2^_G_ = 0.109), followed by 1M2F (*F* (1, 23) = 63.03, *P* < 0.0001, η^2^_G_ = 0.076), 2M1F (*F* (1, 11) = 55.96, *P* < 0.0001, η^2^_G_ = 0.053), 1M1F (*F* (1, 11) = 18.73, *P* = 0.001, η^2^_G_ = 0.037) and 3 F (*F* (1, 11) = 21.77, *P* = 0.0007, η^2^_G_ = 0.018) (Fig. 1).

The results of the first experiment generally showed that the presence of co-actors with each gender composition can induce the social facilitation effect on RT when performing visual search tasks. Four rules in the priority order of strong-to-weak intensity of social facilitation effect were obtained. First, the intensity of the social facilitation effect was stronger when at least one opposite-sex co-actor was present than when all co-actors were same-sex. Second, in the mixed-sex co-action condition, the larger the number of co-actors, the stronger the social facilitation effect was. Third, the social facilitation effect was stronger in the mixed-sex co-action condition than in the all same-sex co-action condition or all opposite-sex co-action condition. At last, when only opposite-sex co-actors were present, the presence of more co-actors resulted in stronger social facilitation effect.

### Experiment 2: Opposite-sex Co-action Results in Greater Arousal than Same-sex Co-action Does

#### Behavioural measures

**Figure 2:**
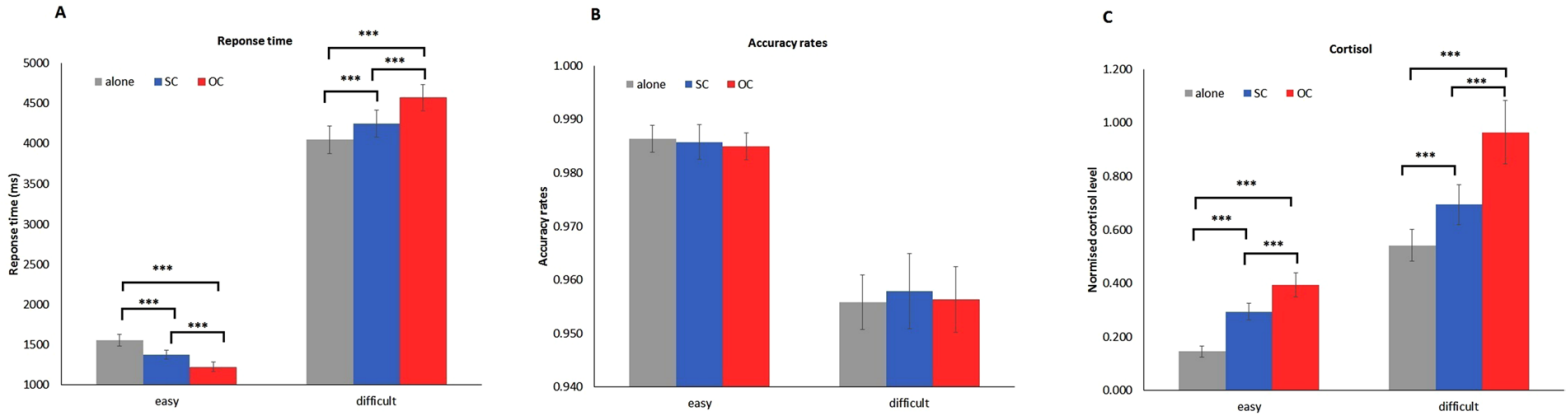
(**A**) RT as a function of task difficulty and social presence (Experiment 2). (**B**) AR as a function of task difficulty and social presence (Experiment 2). (**C**) Normalised cortisol level as a function of task difficulty and social presence (Experiment 2). Error bars are ± 1 SE. ***P < 0.0001,**P < 0.001, *P < 0.05.

In accordance with the results of Experiment 1, when participants performed easy modular arithmetic tasks, they responded faster in the presence of an OC and an SC than in the working-alone condition (*M*_alone_ = 1,550, *M*_SC_ = 1,370, *t* (30) = 6.32, *P* < 0.0001; *M*_alone_ = 1,550, *M*_OC_ = 1,219, *t* (30) = 10.74, *P* < 0.0001); RT was shorter in the presence of an OC than in the presence of an SC (*t* (30) = 9.88, *P* < 0.0001) (Fig. 2A). For difficult tasks, the presence of an OC and an SC both lengthened the RT in contrast to working alone (*M*_alone_ = 4,047, *M*_SC_ = 4,251, *t* (30) = 6.24, *P* < 0.0001; *M*_alone_ = 4,047, *M*_OC_ = 4,573, *t* (30) = 9.76, *P* < 0.0001); RT was larger in the presence of an OC than in the presence of an SC (*t* (30) = 6.78, *P* < 0.0001) (Fig. 2A). Therefore, the presence of an SC or an OC could both induce the social facilitation effect, and the intensity of the social facilitation effect on RT was stronger in the opposite-sex co-action condition than in the same-sex co-action condition, which are consistent with the findings in Experiment 1. The effect of social presence (*F* (1.68, 50.40) = 0.12, *P* = 0.89) and the interaction between task difficulty and social presence (*F* (1.7, 51.8) = 0.14, *P* = 0.87) on ARs were insignificant (Fig. 2B). Thus, the social facilitation effect induced by the presence of co-actors acted on RT rather than on ARs.

#### Normalised cortisol level

The normalised cortisol level was significantly influenced by the task difficulty (*F* (1, 30) = 65.54, *P* < 0.0001), social presence (*F* (1.22, 36.54) = 33.14, *P* < 0.0001) and the interaction between task difficulty and social presence (*F* (1.26, 37.74) = 5.24, *P* = 0.008). Difficult tasks resulted in higher normalised cortisol level than easy tasks did (*M*_difficult_ = 0.733, *M*_easy_ = 0.278, *t* (30) = 8.10, *P* < 0.0001). The normalised cortisol level was highest in the condition of working with an OC, lower in the condition of working with an SC and lowest in the condition of working alone (*M*_alone_ = 0.343, *M*_SC_ = 0.494, *t* (30) = 6.89, *P* < 0.0001; *M*_alone_ = 0.343, *M*_OC_ = 0.680, *t* (30) = 6.30, *P* < 0.0001; *M*_SC_ = 0.494, *M*_OC_ = 0.680, *t* (30) = 4.36, *P* < 0.0001) (Fig. 2C).

#### EEG recordings

**Figure 3:**
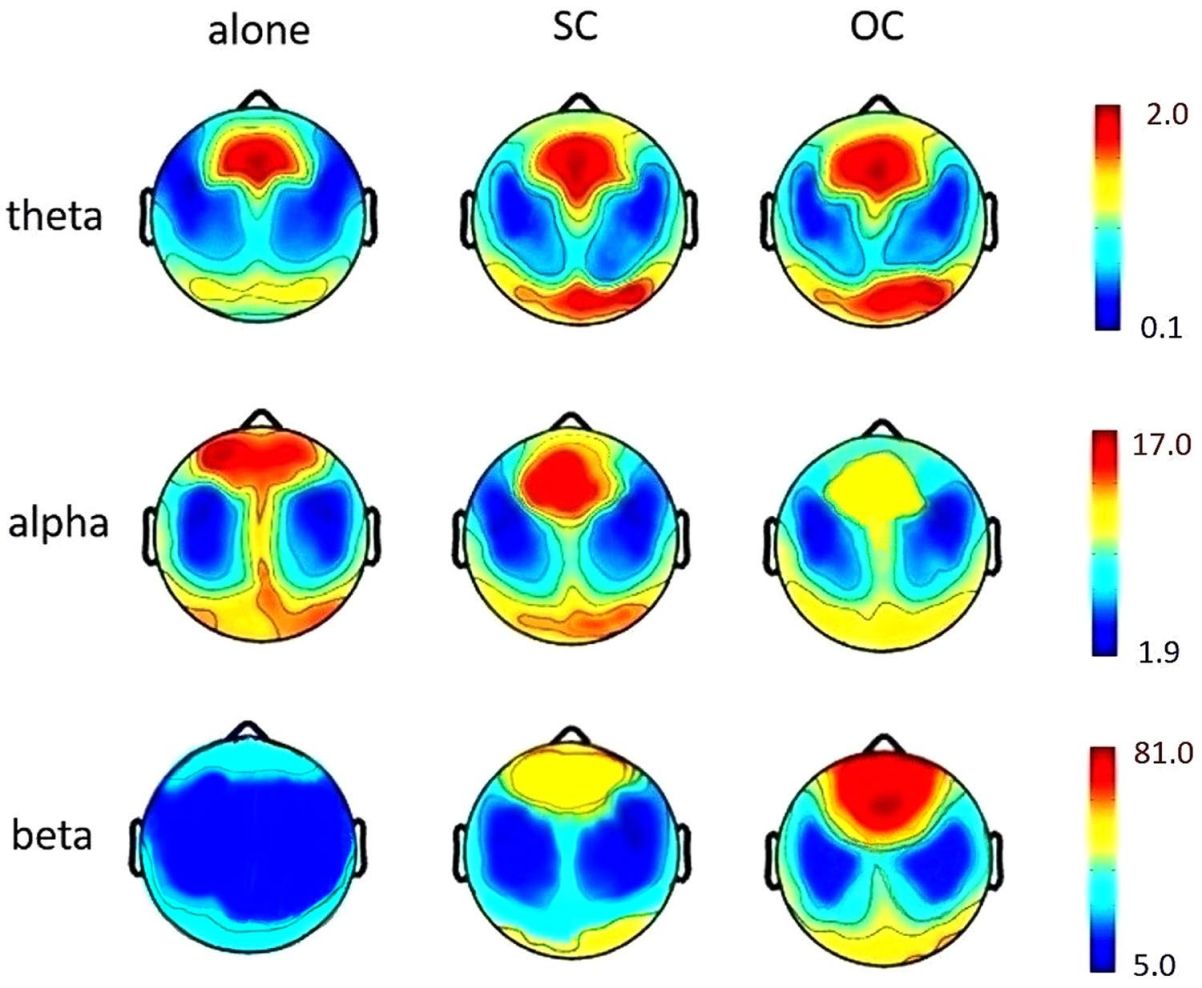
Topographical distribution of theta, alpha and beta power in three social presence conditions. Plots were based on the power spectra of all epochs starting from the onset of the problem statement presented on the screen to the pressing of the P or Q key on the keyboard. Plots were averaged for each social presence condition. Numbers marked on each scale bar indicated the maximal and minimal values (in square microvolts) for theta, alpha, and beta band respectively. Higher values were coded in red and lower values in blue. The prominently activated areas were frontal, parietal and occipital lobes.

The topographical distributions of theta, alpha and beta power in different social presence conditions were presented in Fig. 3. The prominently activated areas were frontal, parietal and occipital lobes, which were consistent with previous findings that frontal, parietal and occipital lobes were involved in numerical computation^51–53^.

**Figure 4:**
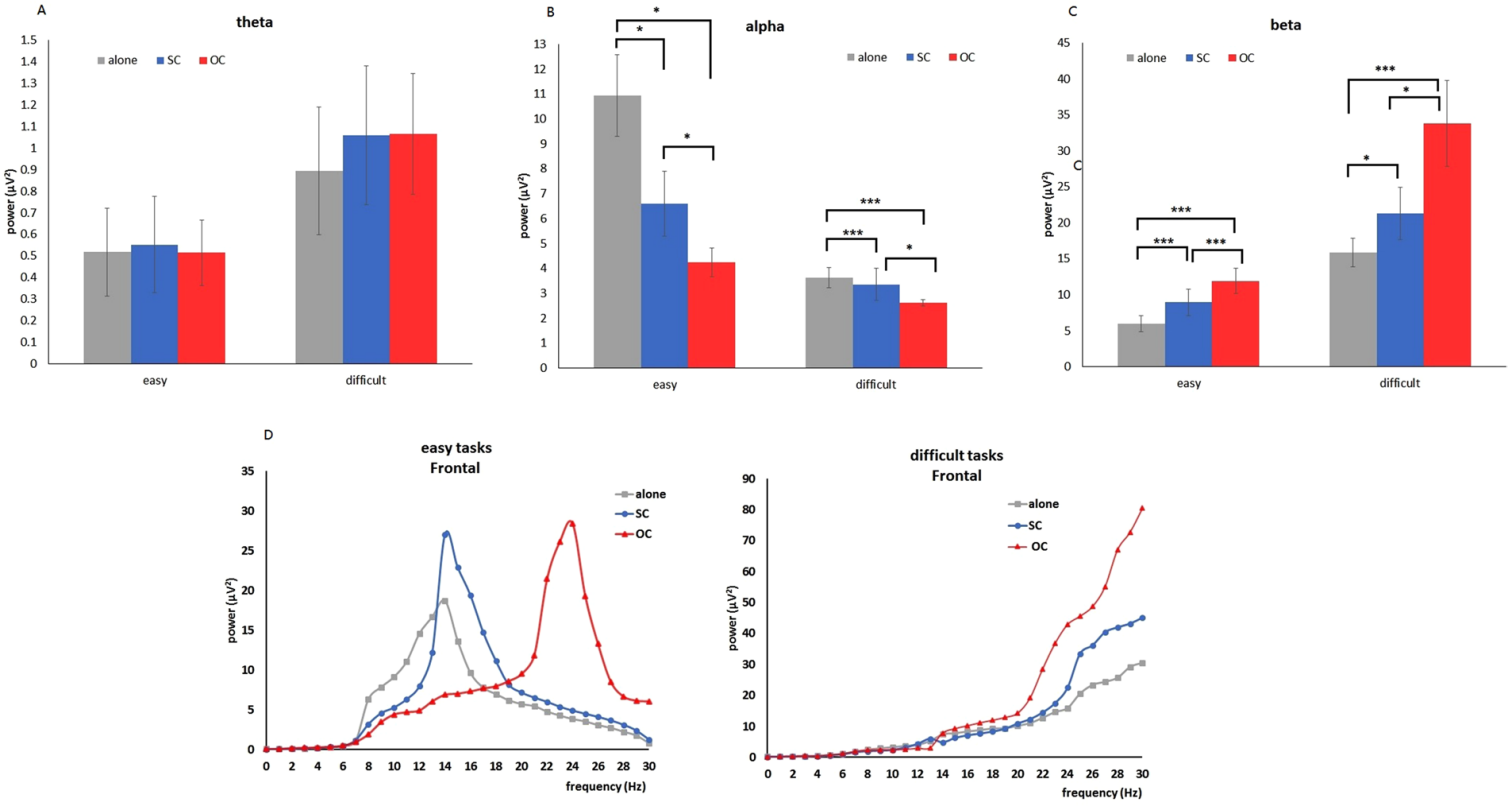
(**A**) Power of the theta band in the frontal lobe as a function of task difficulty and social presence. (**B**) power of the alpha band in the frontal lobe was smallest in the presence of an OC, larger in the presence of an SC, and largest in working alone condition. (**C**) power of the beta band in the frontal lobe was largest in the presence of an OC, smaller in the presence of an SC, and smallest in working alone condition. (**D**) Changes in EEG spectral power in the frontal lobe. Social presence (working alone, working with an SC, and working with an OC) induced changes in EEG spectral power during easy tasks and difficult tasks. Error bars are ± 1 SE. ***P < 0.0001, **P < 0.001, *P < 0.05.

The power of theta band was significantly influenced by task difficulty in the frontal and parietal lobes (frontal: *F* (1, 30) = 60.77, *P* < 0.0001; parietal: *F* (1, 30) = 31.68, *P* < 0.0001). The power of the theta band in the frontal lobe was higher in difficult search tasks than in easy search tasks (Fig. 4A), which was also true in parietal lobe. The main effect of social presence (frontal: *F* (2, 60) = 1.09, *P* = 0.34; temporal: *F* (2, 60) = 2.91, *P* = 0.06; central: *F* (2, 60) = 1.85, *P* = 0.17; parietal: *F* (2, 60) = 2.72, *P* = 0.07; occipital: *F* (2, 60) = 1.64, *P* = 0.20) and the interaction between task difficulty and social presence (frontal: *F* (2, 60) = 0.93, *P* = 0.40; temporal: *F* (2, 60) = 0.01, *P* = 0.99; central: *F* (2, 60) = 0.09, *P* = 0.91; parietal: *F* (2, 60) = 0.38, *P* = 0.69; occipital: *F* (2, 60) = 0.15, *P* = 0.86) on the power of the theta band in all of the lobes were insignificant.

With regard to the power of the alpha band, the main effects of task difficulty (frontal: *F* (1, 30) = 16.04, *P* = 0.0004; parietal: *F* (1, 30) = 28.43, *P* < 0.0001; occipital: *F* (1, 30) = 18.18, *P* = 0.0002), social presence (frontal: *F* (1.19, 35.58) = 10.17, *P* = 0.0002; parietal: *F* (1.35, 40.38) = 15.54, *P* < 0.0001; occipital: *F* (1.21, 36.24) = 10.17, *P* = 0.0001) and the interaction between task difficulty and social presence (frontal: *F* (1.20, 36.06) = 4.56, *P* = 0.014; parietal: *F* (1.50, 44.88) = 6.23, *P* = 0.003; occipital: *F* (1.27, 38.04) = 5.68, *P* = 0.006) in the frontal, parietal and occipital lobes were significant. In the frontal lobe, alpha power was lower in difficult tasks than in easy tasks (Fig. 4B). The presence of a co-actor reduced the alpha power compared with the condition of working alone; and the alpha power was higher in the presence of an SC than in the presence of an OC (Fig. 4B) (in easy tasks: *M*_alone_ = 11.0, *M*_SC_ = 6.5, *t* (30) = 2.27, *P* = 0.03, *M*_alone_ = 11.0, *M*_OC_ = 4.3, *t* (30) = 3.02, *P* = 0.005, *M*_SC_ = 6.5, *M*_OC_ = 4.3, *t* (30) = 2.93, *P* = 0.006; in difficult tasks: *M*_alone_ = 3.4, *M*_SC_ = 2.5, *t* (30) = 5.61, *P* < 0.001, *M*_alone_ = 3.4, *M*_OC_ = 1.7, *t* (30) = 4.34, *P* < 0.001, *M*_SC_ = 2.5, *M*_OC_ = 1.7, *t* (30) = 2.69, *P* = 0.012). Similar results were obtained in the parietal and occipital lobes.

On the power of the beta band, task difficulty (*F* (1, 30) = 33.33, *P* < 0.0001), social presence (*F* (1.20, 36.06) = 22.97, *P* < 0.0001) and their interaction (*F* (1.33, 39.96) = 7.64, *P* = 0.001) significantly affected the frontal lobe. Difficult tasks induced larger beta power than easy tasks. Beta power was strongest in the condition of working with an OC, followed by the condition of working with an SC and the condition of working alone (Fig. 4C) (in easy tasks: *M*_alone_ = 5.9, *M*_SC_ = 8.9, *t* (30) = 3.91, *P* < 0.001, *M*_alone_ = 5.9, *M*_OC_ = 12.8, *t* (30) = 4.50, *P* < 0.001, *M*_SC_ = 8.9, *M*_OC_ = 12.8, *t* (30) = 4.21, *P* < 0.001; in difficult tasks: *M*_alone_ = 15.9, *M*_SC_ = 20.9, *t* (30) = 3.11, *P* = 0.004, *M*_alone_ = 15.9, *M*_OC_ = 33.3, *t* (30) = 4.46, *P* < 0.001, *M*_SC_ = 20.9, *M*_OC_ = 33.3, *t* (30) = 3.81, *P* = 0.001).

In summary, significant effects were only observed in the frontal lobe for all three band powers, i.e., theta, alpha and beta. Figure 4D illustrates the changes in the EEG spectral power in the frontal lobe. The results of correlational analyses indicated that the power of theta (Pearson *r* = 0.214, *P* < 0.01) and beta bands (Pearson *r* = 0.341, *P* < 0.001) in the frontal lobe were positively correlated to normalised cortisol level, and the alpha power in the frontal band was negatively correlated to normalised cortisol level (Pearson *r* = −0.269, *P* < 0.001).

## Discussion

In the present study, the presence of SCs can induce the social facilitation effect when actors perform visual search tasks and modular arithmetic tasks, which is similar to the findings of previous studies^44,45^. Moreover, the presence of an OC could induce a stronger social facilitation effect than the presence of an SC. In addition, the intensity of the social facilitation effect in varying gender compositions of co-actors has also been examined and compared. More importantly, the present study is the first to measure an actor’s arousal level in the working-alone condition and co-action condition by using physiological indicators (i.e., EEG activity and salivary cortisol). The common key point of previous social facilitation theories is that people exhibit greater arousal in the presence of others than alone^7,8,12,25^. The current study validated their shared argument. Furthermore, the obtained results indicated that OC could induce a higher arousal level of participants than SC and working alone. Consistent with prior findings^1,54,55^, the social facilitation effect only existed in RT rather than ARs. In the study of Liu and Yu (2017), eye movements were utilized to explore why the social facilitation effect evoked by the presence of others did not exist in AR when people performed visual search tasks. Their results showed that the unchanged accuracy resulted from the unchanged target fixation numbers in the two social presence conditions for both easy and difficult settings. The visual search tasks utilized in the present study were the same as those in their study^55^. Thus, it could be inferred that the social facilitation effect induced by the presence of co-actors did not influence the accuracy rates because the target fixation number did not change significantly between alone condition and coaction condition. As a result, the response time could be more reliable in measuring performance variations in response to social presence than accuracy rates in visual search tasks, as previous literature review has claimed^6,56^.

Previous studies suggest that individuals often control their behaviour in front of opposite-sex people^57,58^ as they are particularly concerned about managing their impressions when interacting with the opposite-sex^59^. Their self-presentational motivations are stronger in interactions with unfamiliar people of the opposite-sex than in interactions with unfamiliar people of the same-sex, and no gender difference exists between males and females in terms of making impressions on others^60^. All of these findings were supported by the evidence of EEG activity and salivary cortisol levels obtained in the present study. The power of the alpha band was larger in the condition of working with an SC than in the condition of working with an OC whereas the opposite is true for the power of the beta band and the normalised cortisol level. Alpha power is negatively correlated with arousal level^33,36,37^, whilst beta power and salivary cortisol are positively linked to arousal^39,40,42,43^. Thus, the results demonstrate that an OC can induce higher arousal level among participants, thereby resulting in greater social facilitation effect compared with an SC, because the presence of an OC can result in more self-presentation motives among actors in contrast to an SC.

The results of the present study show that the social facilitation effect was accompanied by reduced activity of the alpha band in the frontal, parietal and occipital lobes, as well as enhanced activity of the beta band in the frontal lobe. The frontal and parietal areas, which are associated with attention^61,62^, exhibit changes in EEG activities in relation to social presence. Attention enables us to select relevant information for further processing whilst ruling out other irrelevant distractors^63,64^. The presence of others creates an attentional conflict arising from the intention of individuals to concentrate both on the task and others at the same time^65^. The attentional conflict distracts individuals when they perform tasks, thereby prompting individuals to focus their attention on avoiding the distraction^13,65^, and the attentional conflict is a source of increased arousal^65^. The results of the present study show that the presence of co-actors enhanced EEG activity in areas associated with attention, which is consistent with distraction–conflict theory^12^, i.e., social presence intensifies the demands for filtering distractions. Similarly, a study of primates revealed that when another monkey was present, the performance of the experimental money was enhanced, and the activity in the attention frontoparietal network encompassing the lateral prefrontal cortex, ventral premotor cortex, frontal eye field and intraparietal sulcus, increased^66^.

Some studies claimed that crowding (i.e., increase in group size or density) can have negative psychological effects on humans^67,68^, whereas other studies found that crowding can have positive effects^69^. Considering the findings in the present study, the effect of group size on the performance of individuals was mediated by task difficulty; that is, in easy tasks, crowding produces positive effects and enhances individual performance, whereas in difficult tasks, crowding results in negative effects and impairs individual performance. Furthermore, the results suggest that the gender composition of co-actors in a group could modulate the intensity of social facilitation effect. In contrast to the same-size group consisting of all SCs and all OCs, the group consisting of mixed-sex co-actors had stronger social facilitation effect. This result might be attributed to the synergy effects resulting from the self-presentation motives of individuals in front of people of the opposite sex^60^ and the comparison to same-sex others^70–72^. Therefore, future studies should explore the underlying causes and mechanism in this condition.

Theta power increases as task workload increases and is most evident in frontal areas^73,74^. In addition to the theta band, changes in EEG activity related to increased task workload can also be observed in the alpha band. A decrease in alpha power is associated with increased task requirements^73,75^. The present results demonstrate that as task difficulty increases, the power of the theta band in the frontal and parietal lobes increases, whereas the power of the alpha band decreases, which is consistent with previous findings.

Hypothalamus–pituitary–adrenocortical axis can be activated to release cortisol in response to stress and anxiety^41,76,77^. High cortisol levels are associated with high stress, anxiety and arousal^41–43^. Anxiety is a negative emotional state in which feelings of nervousness, worry and apprehension are associated with individual arousal^21^, and stress is the non-specific response of a human organism to any demand placed upon it^78^. If an individual is unfamiliar with the other person present, they would feel uncertain and anxious^22,23^. In the presence of co-actors, individuals are afraid of being evaluated by others present^24,25^. As a result, the sense of uncertainty and apprehension brought about by the presence of co-actors might result in the individual’s stress and anxiety. The results reveal that cortisol levels were higher in the presence of co-actors than when working alone. Thus, the present study verifies previous social facilitation theories from the physiological perspective. In addition, the salivary cortisol level was higher in the presence of an OC present than when an SC is present. This finding is consistent with previous findings which claim that cortisol levels are significantly improved when males are exposed to females^79,80^.

While the findings here should benefit managers and researchers involved in work organization, a limitation should be noted. Based on the results of behavioural performance in Experiment 1, some rules concerning the intensity of social facilitation effect in different co-action conditions were proposed. However, Experiment 2 only measured the arousal level of three conditions (i.e., alone, with a SC and with an OC), and provided physiological arousal evidence for the stronger social facilitation effect induced by OC than SC as indicated in Experiment 1. The other conditions pertaining to varying gender composition of co-actors were not involved and need to be examined in future research.

The results of the present study demonstrate that working with various gender compositions of co-actors can induce the social facilitation effect for actors when performing visual search tasks and modular arithmetic tasks. The mediating effect of gender compositions of co-actors on the intensity of the social facilitation effect has also been examined and compared. The social facilitation effect was generally stronger in the presence of OCs than in the presence of all SCs both for males and females. Mixed-sex co-actors elicited stronger social facilitation than OCs and SCs. On the other hand, this study was the first to test from the physiological perspective whether the presence of others can increase individuals’ arousal. EEG activities and salivary cortisol levels both indicate that the presence of a co-actor could increase the arousal level of actors. The neurophysiological evidence is consistent with social facilitation theories proposed from psychological aspects whose explanation suggests that increased arousal leads to the social facilitation effect. Therefore, these findings provide insights into the influencing mechanisms of gender composition on the intensity of the social facilitation effect as well as implications for selecting and organising people of different genders in co-action conditions.

## Materials and Methods

### Participants

Both experiments were approved by Institutional Review Board of Tsinghua University and conducted in accordance with the approved human ethics guidelines. All participants were informed of the instructions and procedure of experiments and signed written informed consent forms prior to the experiments. In Experiment 1, 144 undergraduates aged 17 to 23 (72 males and 72 females) were recruited as participants (*M* = 19.7, *SD* = 1.2). All of the participants had normal or corrected normal visual acuity. None of the participants had any experience with similar visual search tasks.

In Experiment 2, 32 male graduates aged 22 to 30 (*M* = 25.4, *SD* = 2.2) were recruited to participate. One participant was run in Experiment 2 but excluded later on because of excessive artifacts in the EEG (>30% of EEG data had to be excluded due to muscle movement artifacts). Thus, Experiment 2 had 31 valid participants. All participants were right-handed. Similar to previous studies, the same persons were used as the confederates of the experimenter in the present study because of its ease of controlling and manipulating^81,82^. Two research assistants (RAs, one male and one female) were recruited as pseudo participants to act as the SC and OC respectively in order to avoid the effects of some covariates, such as attractiveness and social comparison^83,84^. Thus, each co-action condition had one actual participant and one pseudo participant. Actual participants were unaware that their co-actors were RAs.

### Apparatus and software

The software used in Experiments 1 and 2 were developed by C# language on Microsoft Windows platform (Microsoft, United States) to present the stimuli and record performance. They were run on an Acer Aspire desktop (Intel Core i5-6400, Windows 10) with a 21.5-inch Acer S220HQL LCD monitor (1,920 × 1,080 pixel resolution, 70-Hz refresh frequency). Participants sat on a chair with an adjustable height in front of the display from a viewing distance of 60 cm controlled by using a chin rest. The adjustable chair was used to ensure that the participants’ eyes were roughly perpendicular to and at the centre of the display. Participants performed experimental tasks by using a mouse and a keyboard.

**Figure 5:**
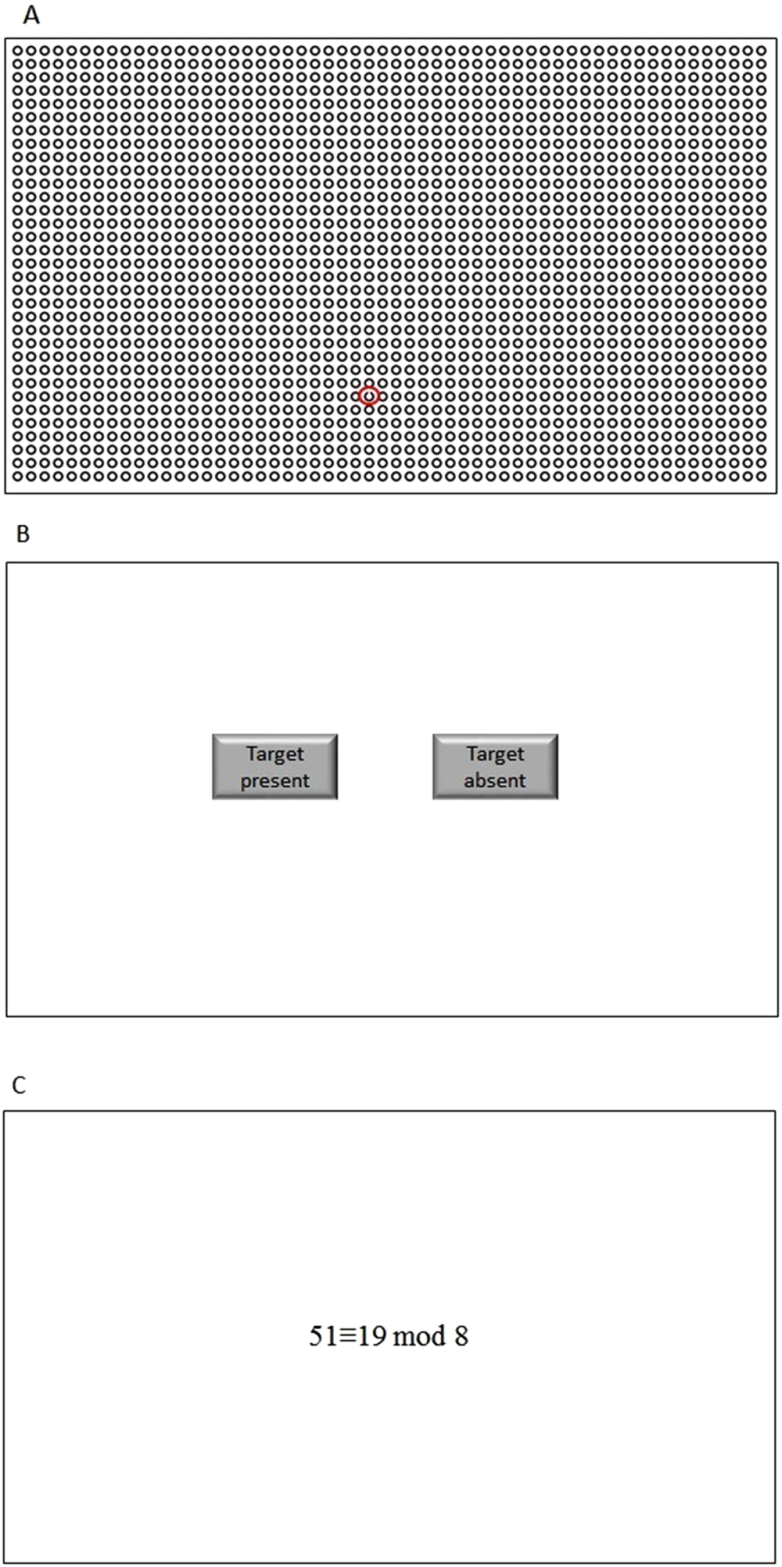
(**A**) The stimuli presentation interface of difficult visual search tasks (the target is circled) in Experiment 1. (**B**) the response selection interface in Experiment 1. (**C**) an experimental interface sample of difficult trials in Experiment 2.

*Experiment 1*. The software in Experiment 1 had three types of interfaces, i.e., initial interface, stimuli presentation interface and response selection interface. In the initial interface, a fixation cross was presented at the centre of the interface. The stimuli presentation interface had a size of 1,120 × 660 pixels, subtending visual angles of 28.9° horizontally and 17.2° vertically to participants’ eyes. The interface was used to present the search stimuli (Fig. 5A). The response selection interface had two buttons (i.e., target present and target absent) (Fig. 5B). Participants responded to target presence in this interface by clicking corresponding buttons.

*Experiment 2*. The software in Experiment 2 had one interface to present the problem statement at the centre of the screen, e.g., 51 ≡ 19 mod 8 (Fig. 5C). Participants were asked to decide whether the statement was true or false by pressing the corresponding key (Q for true and P for false) on the keyboard.

### Stimuli

*Experiment 1*. The stimuli consisted of several homogeneous distractors in the background and at most, one target among the distractors. Both target and distractor images had a square size of 20 × 20 pixels, subtending visual angles of 0.52° horizontally and vertically to participants’ eyes. The target images were black standard Landolt Cs with four orientations (i.e., up, down, left and right). The type of target images was randomly selected by the software. The distractor images were complete Os. In target-present search trials, only one search target required participants to search, and the target image randomly appeared in any place in the search area. To avoid exceptionally long and short RTs, the search targets did not appear in the marginal and central positions of the search area. The task difficulty was controlled by the number of distractors in the stimuli^85^. In difficult visual search tasks, images filled in all of the grids of the stimuli. In easy visual search tasks, only the grids in the odd column and odd row contained images. The total number of images in the difficult search tasks was 1,848 (56 columns × 33 rows), which was twice as that in the easy search tasks.

*Experiment 2*. This experiment had one experimental interface to present the problem statement that asked participants to evaluate whether the statement is true or false. The problem statement consisted of three numbers, and the computation rule was as follows. Taking 51 ≡ 19 mod 8 as an example, the statement’s middle number is subtracted from the first number (i.e., 51−19) at first, and the result (i.e., 32) is divided by the last number (i.e., 32 ÷ 8). If the quotient is a whole number (in this case, 4), then the problem statement is true. If the quotient is not a whole number, then the problem statement is false. The task difficulty was manipulated by controlling the number of digits presented to participants for the first two numbers of a given problem; one for an easy task (7 ≡ 2) and two for a difficult task (51 ≡ 19)^5^.

### Procedure

*Experiment 1*. The experiment consisted of five sessions: one practice and four actual experimental sessions. The tasks used in the practice session were the same as those in the actual experimental sessions. Participants conducted the practice and actual experimental sessions on two consecutive days. On the first day, participants were required to finish the practice session. Upon arrival at the laboratory, participants were given an introduction of the experiments and signed informed consent forms. In the practice session, participants were provided with instructions on how to conduct the visual search tasks and use the software. After participants were familiarised with the software, they were required to complete both easy and difficult search tasks individually, and their performance measures were recorded. Participants were assigned to one of the six co-action groups according to the gender composition and the performance level of each participant, i.e., participants with similar performance levels were assigned to the same group to eliminate the possible effect of social comparison on the experiment^86,87^, which should also meet the requirements of the gender composition. All of the participants were not familiar with each other in each co-action condition. In the end, the 1M2F group consisted of 12 males and 24 females, and the 2M1F group consisted of 24 males and 12 females. A total of 12 males were assigned to the 3 M group and 12 females were assigned to the 3 F group. The 1M1F and 2M2F both included 12 males and 12 females in each group.

On the second day, participants were required to finish four actual experimental sessions. The four sessions corresponded to the four combinations of task difficulty and social presence, i.e., performing easy search tasks alone, performing difficult search tasks alone, performing easy search tasks in the presence of co-actors and performing difficult search tasks in the presence of co-actors. The orders of the four combinations were counterbalanced across participants. Each session had 60 search trials, 30 of which were target-present trials and 30 were target-absent trials. All of the trials in each session were sorted randomly. Participants needed to finish a total of 240 visual search trials (60 trials × 4 sessions) in the actual experiment. After finishing each session, participants had a 15-min rest.

**Figure 6:**
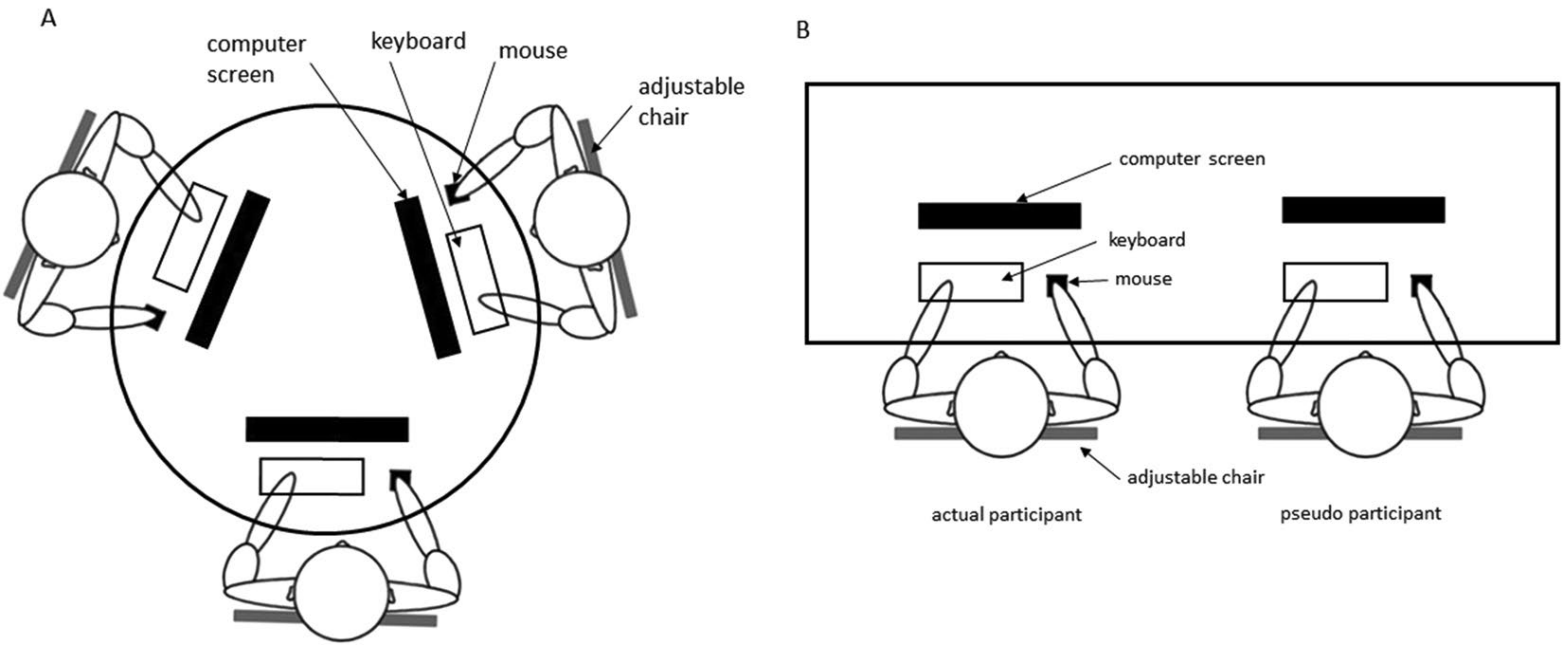
(**A**) Experimental setup for three co-actor conditions in Experiment 1. All participants were actual participants. (**B**) Experimental setup in the condition of working with an SC or an OC in Experiment 2. One actual participant performed tasks in the presence of a same-sex pseudo participant or an opposite-sex pseudo participant.

During the actual experiment, participants were instructed to search in the stimuli presentation interface and to respond to the target presence as quickly and as accurately as possible. In the working-alone condition, participants were required to perform the visual search tasks on their own. In the condition of working with co-actors, two to four participants were seated around a table with an equal 90-cm distance and performed visual search tasks independently (Fig. 6A). Participants were informed that they only performed the visual search tasks with others at the same table but that they were not in a competition and their performances were not compared. The experimenter then left the laboratory, and the participants began their own actual experimental sessions.

In each visual search trial, participants were asked to gaze at the fixation cross at the centre of the screen^88^ and to click on the cross to enter the stimuli presentation interface. When participants finished one search trial (i.e., they were able to give their response of target presence), they pressed the space key on the keyboard to end the current search trial. After participants pressed the space key, the response selection interface replaced the stimulus presentation interface with two buttons appearing on the screen, and the participants needed to give their responses of target presence. If the participant thought that the previous stimuli presentation interface contained a target, he used the mouse to click the target-present button to declare that he had found the target. If the participant felt that no target existed in the previous stimuli presentation interface, he used the mouse to click the target-absent button to report that the target was absent. Regardless of their response, the clicking of the target-present or target-absent button would display the fixation cross on the screen again and let participants take the next trial. The same procedure was repeated until all of the actual experimental trials were finished.

*Experiment 2*. This experiment included one practice session and six actual experimental sessions. The modular arithmetic tasks in the practice session were the same as those in the actual experimental sessions. Upon arrival at the laboratory, participants were given an introduction to the experiments and signed informed consent forms. They provided the first saliva sample to train the participants on how to collect saliva samples^42^. Thereafter, they were required to perform tasks in the practice session, i.e., complete at least 30 easy and 30 difficult modular arithmetic trials to familiarise themselves with the procedure and instructions. After completing the practice session, participants had a 15-min rest before the actual experimental trials. Their EEG activity during the practice session was not recorded.

In the actual experimental sessions, participants were asked to finish six sessions corresponding to the six combinations of task difficulty and social presence, i.e., perform easy and difficult modular arithmetic trials in the condition of working alone, in the presence of an SC and in the presence of an OC. The orders of the six combinations were counterbalanced across participants. Each session had 200 trials, 100 of which were true trials and 100 were false trials. All of the trials in each session were sorted randomly. Participants needed to finish a total of 1,200 trials (200 trials × 6 sessions) in the actual experiment. Participants simultaneously performed modular arithmetic tasks with their EEG recorded. After completing each session, the participants had a 20-min rest and provided a saliva sample at the end of the rest, because the time latency to reveal a psychological stressor effect by salivary cortisol is approximately 15–20 min^89^. Six saliva samples (S_1_–S_6_) corresponding to six combinations were obtained from each participant at the end of the experiment. In each modular arithmetic trial, a problem statement, such as “51 ≡ 19 mod 8”, appeared on the screen. Participants were asked to decide whether the statement was true or false by pressing the corresponding key (Q for true and P for false) on the keyboard.

In the working-alone condition, participants were required to perform the modular arithmetic tasks on one’s own. In the condition of working with co-actors, a male and a female RA were asked to sit at the same table next to the actual participant at a distance of 90 cm and served as pseudo participants (Fig. 6B). RAs and participants did not know each other. One actual participant and one pseudo participant performed the modular arithmetic tasks independently without any communication in the condition of working with an SC and an OC. The EEG data of pseudo participants were not analysed. Before the experiment, the pseudo participants already knew the purpose of the experiment and were familiarised with the software. In addition, they were required to end the session when the actual participants finished the session to reduce the influences of social comparison^86,87^. To avoid the sense of competition for the actual participants, all of the participants were informed that they only performed the visual search tasks with the other person at the same table but that they were not in a competition and their performances were not compared. Only after actual participants finished each session could the experimenter return to the laboratory to collect the salivary sample and set the software for the next session. When the participants finished all of the experimental sessions, they left the laboratory. They were asked to collect one more salivary sample at home on another day at the same time of day that they had been in the laboratory, to obtain a better baseline sample in a stress-free setting^42^. These after-experiment assessments of salivary samples (S_0_) were used as the cortisol baseline measures.

### Measures

*Experiment 1*. The search performance was measured by RT and ARs. In this experiment, RT refers to the time interval between the start of the visual search (i.e., clicking on the fixation cross) and the end of the search (i.e., pressing of the space key) in each trial. AR refers to the percentage of the number of correct response trials divided by the total number of trials.

*Experiment 2*. In this experiment, RT refers to the time interval between the onset of the experimental interface and the pressing of Q or P on the keyboard. AR refers to the percentage of the number of correct response trials divided by the total number of trials. Salivary cortisol level was normalised to the baseline measure, i.e., calculating the rate of change between the cortisol level of the salivary samples collected during experiments and the baseline cortisol measure (i.e., [cortisol level of S_i_−cortisol level of S_0_]/cortisol level of S_0_, i = 1, 2, 3, 4, 5, 6).

### Data analysis

All of the statistical analyses were performed by SPSS Statistics 20.0 (IBM, United States). An α of 0.05 was used for all statistical tests. Paired *t* tests with Bonferroni adjustment were adopted for post hoc analysis. EEG data analysis was performed with MATLAB R2012a (MathWorks, United States) including EEGLAB toolbox^90^.

*Experiment 1*. Mixed-model analyses of variance (ANOVAs) were used to determine the main and interaction effects of task difficulty, social presence, gender and gender composition on performance variables. Effect size was calculated with a generalized eta-squared (η^2^_G_)^91^.

*Experiment 2*. Repeated-measure ANOVAs were used to determine the main and interaction effects of task difficulty and social presence on dependent variables. For within-subject variables, Mauchly’s test was used to test the sphericity assumption. In case the sphericity assumption was violated, the degrees of freedom were corrected using Greenhouse–Geisser estimates of sphericity. The correlational analyses on EEG recordings and normalised cortisol level were performed by calculating Pearson *r*.

### EEG recordings and analysis in Experiment 2

The EEG activity was recorded using 32 Ag/AgCl electrodes mounted in an elastic cap placed at the scalp and amplified by a NuAmps amplifier (Neuroscan, United States). Electrodes were positioned according to the international 10–20 system and were referenced to linked mastoids (average of A1 and A2) offline, and a ground electrode was located in the forehead. The sampling rate and filter bandwidth were set to 500 Hz and 1–100 Hz, respectively. All of the electrode impedances were kept below 5 kΩ for the EEG. The EEG activity data were filtered offline with an analogue bandpass filter of 0.01–100 Hz. Electrooculogram (EOG) activity was also recorded from two additional bipolar channels to facilitate subsequent EEG artifact rejection. The EOG was corrected offline using an independent component analysis method^92^. Other aberrant signals exceeding a 100μV threshold were discarded^93^. Artifact rejection was performed for each trial on epochs starting from the onset of the problem statement presented on the screen to the pressing of the P or Q key on the keyboard.

Artifact-free EEG data were grouped into 30-s epochs and a fast Fourier transformation was applied to each epoch with a non-overlapped Hanning window of 1 s. The power spectra from each epoch were then averaged to yield average power spectra for each trial at all electrodes. Spectra were grouped into three frequency bands: theta (θ: 4–7 Hz), alpha (α: 8–13 Hz) and beta (β: 14–30 Hz)^94^. In this study, the alpha, theta and beta bands of EEG data in frontal, temporal, central, parietal, and occipital lobes were analyzed and compared separately. The power of theta, alpha and beta on each combination of task difficulty and social presence in each lobe were the average power spectra for all trials corresponding to different electrodes. The frontal lobe included F7, F3, Fz, F4 and F8 electrodes. The temporal lobe corresponded to T3, T4, T5 and T6 electrodes. The central lobe corresponded to C3, Cz and C4 electrodes. P3, Pz and P4 were the electrodes for the parietal lobe, and O1, Oz and O2 were the electrodes for the occipital lobe.

### Saliva collection and assays in Experiment 2

Saliva was collected using Salivette (Sarstedt AG & Co., Germany). Participants placed the swab from the Salivette into the mouth and chewed it for about 60 s and then returned the swab with the absorbed saliva to the Salivette. All salivary samples were stored frozen at −20 °C until analysis. Salivary cortisol levels were assayed by using commercial enzyme-linked immunosorbent assay (ELISA) kits (salivary cortisol ELISA, SLV-2930, DRG Instruments GmbH, Germany) with a sensitivity of 0.537 ng/mL. The assay of each sample was replicated twice and only measures whose coefficient of variation was lower than 10% were used (intra-assay of 2.7% and interassay coefficient of variation of 5.7%).

### Data Availability

All data generated or analysed during this study are included in this article.
